# Fifty-Five Years of Research on *B*, *C* and *D* in *Escherichia coli*

**DOI:** 10.3390/life13040977

**Published:** 2023-04-10

**Authors:** Charles E. Helmstetter

**Affiliations:** Department of Biomedical and Chemical Engineering and Sciences, Florida Institute of Technology, Melbourne, FL 32901, USA; chelmste@fit.edu

**Keywords:** cell cycle, synchronous cells, chromosome replication, initiation age, *B* period, *C* period, *D* period

## Abstract

The basic properties of the *Escherichia coli* duplication process can be defined by two time periods: *C*, the time for a round of chromosome replication, and *D*, the time between the end of a round of replication and cell division. Given the durations of these periods, the pattern of chromosome replication during the cell cycle can be determined for cells growing with any doubling time. In the 55 years since these parameters were identified, there have been numerous investigations into their durations and into the elements that determine their initiations. In this review, I discuss the history of our involvement in these studies from the very beginning, some of what has been learned over the years by measuring the durations of *C* and *D*, and what might be learned with additional investigations.

## 1. Introduction

Upon completion of the requirements for a PhD in biophysics at the University of Chicago in 1961, I had the honor to have the diploma handed to me by George Wells Beadle, the newly installed president of the university, recent Nobel laureate and one of the first acclaimed researchers in molecular genetics. The work of Beadle and Edward Tatum, and their demonstration of the one gene–one enzyme hypothesis [1], was a prominent topic in genetics courses at the time. Since that time, I have had the opportunity to read several essays by and about George Beadle and have, as a consequence, become interested in his approach to the study of science. As is described below, my scientific journey followed a similar path, although with a decidedly less consequential outcome. I suspect that many of us may have followed similar paths during the course of our work. However, before discussing that issue, another aspect of Beadle’s thinking about science should be mentioned. He began his contribution to Phage and the Origins of Molecular Biology [2] by writing: “I have often thought how much more interesting science would be if those who created it told how it really happened, rather than reported it logically and impersonally, as they often do in scientific papers. This is not easy, because of normal modesty and reticence, reluctance to tell the whole truth, and protective tendencies toward others”. I have attempted to follow his suggestion in this personal review.

My primary interest in Beadle’s work concerns the story behind his long-term effort to investigate the gene–enzyme paradigm [3,4,5] due to its correspondence with our similar search for a way to determine the relationship between chromosome replication and cell division in *E. coli*. He and Tatum studied the fruit fly, *Drosophila,* in an attempt to understand the gene–enzyme relationship by searching for enzymatic reactions controlled by known genes. This continued for about five years without success. Then, one day, while listening to a lecture by Tatum, he apparently suddenly realized that the best and easiest approach was to conduct the experiments in reverse, that is, to search for genes that controlled known chemical reactions. He realized that experiments of this type could be conducted quite easily by X- or UV-irradiating *Neurospora*, with which he was already familiar, and then by simply selecting and characterizing those mutants that could no longer grow on a minimal medium. It only took 5 months to obtain the first set of nutritional mutants. It was not long before hundreds of mutants with requirements for specific nutrients were generated, thereby producing evidence for the “one gene–one enzyme” hypothesis. Recently, Strauss [5] suggested that the true significance of this work by Beadle and Tatum may be overlooked. They were awarded their Nobel Prize “for their discovery that genes act by regulating definite chemical events”. However, that finding was made possible by Beadle’s simple, but inspired idea to mutagenize, and then select for a required nutrient. It was a revolutionary approach to experimental biology that many in our field have used successfully for decades. As I explain later, our work to identify the *E. coli* replication–division relationship also stemmed directly from a comment made by a colleague, followed by a decision to conduct cell cycle experiments in reverse order.

## 2. Synchronous Cell Growth

### 2.1. Bacterial Cells

My interest in the cell cycle began while I was a graduate student in the mid-1950s, not long after the report by Howard and Pelc defining the stages of the eukaryotic cell cycle was published [6]. I started by performing some radiobiological studies on newt heart cells in mitosis. In an adjacent laboratory, Aaron Novick and Leo Szilard continued to develop and study chemostats [7]. Novick, who had recently returned from the Pasteur Institute, encouraged me to read the magnificent work that had been published by researchers at the Pasteur Institute at that time (e.g., [8,9]). They were the most exciting papers I had read at that young age, and I still feel that way. Between reading about that work and the excitement in the Novick–Szilard lab, I decided to switch my focus to the bacterial cell cycle and study something related to DNA. Based on the thinking at the time, it appeared that the only way to study the cycle was to obtain synchronously dividing cultures. The only bacteria with which I had any familiarity were the strains being used by Novick and Szilard, primarily *E. coli* strain B, and occasionally, B/r. After looking at their cells using a microscope, strain B did not appear to be suitable for synchronization since the size distribution was broad, with many filamentous cells. Strain B/r however looked very promising since the size distribution was narrower, there did not appear to be any filaments, and they were nonmotile. I thought I might be able to synchronize the division of that strain, so I asked my major professor at the time, Robert Uretz, to purchase *E. coli* B/r 12407 from the American Type Culture Collection. That strain was later called B/r A.

Back then, there were basically three different approaches to bacterial synchronization: single or multiple temperature shifts, single or multiple nutritional deprivations, and size selection by filtration or centrifugation [10]. All of these methods were found to cause some growth disturbances, as assessed by the requirements that the cells undergo at least two cycles of detectably synchrony, that whatever is measured in the first cycle repeats in the second cycle, and that the fundamental properties of synchronous cells, such as sizes and growth rates, mimic the initial exponential phase population. The method that appeared to cause the least disturbance involved filtration of a culture through a stack of Whatman cellulose filter papers, which enabled the smaller newborn cells to pass through into the effluent, while retaining the larger, older cells in the stack [11]. A modification of this technique, involving the use of pressure rather than vacuum filtration, enabling me to perform a few simple experiments on *E. coli* B/r A cells at very low concentrations.

Everything changed in 1963 during a meeting of the Biophysical Society in New York. One afternoon, I was involved in a discussion with a small group talking about synchronizing cells. I believe Philip Hanawalt was among the group and am certain another participant was David Friefelder because he asked me a question that completely changed the course of the work. After I described the technique I was using, Friefelder asked a number of questions including how long filtration took. I said, “A few minutes”. He then said, as I recall, “Well then, the cells must be growing while in the filter stack”. That turned out to be the comment that led to the eventual generation of a very simple method to determine the DNA–cell cycle relationship, which did not involve synchronizing cells at all.

The idea was that if growing cells became attached to a substrate while a culture medium passed through, the only cells that could possibly be released from the substrate would be newborn cells originating from the portion of the bound cells that was not involved in their original attachment. Some of the dividing cells might not release a progeny at all, or some progenies might reattach, but in the ideal case in which all attachments are permanent, only newborns will be released. Realistically, strong attachment plus good flushing ought to yield a highly pure population of newborn cells. The best part is that this process might be able to yield a minimally disturbed, synchronously dividing population because it simply involved collecting and incubating cells released from a culture growing under ideal conditions. The theory seemed to work perfectly on the first try. The development and testing of this new approach rapidly progressed, with the final configuration consisting of filtering cells of the strain B/r A using a nitrocellulose membrane filter, inverting the filter, pumping the medium backwards through the filter, and collecting the cells that fell out [12]. It eventually became known as the “baby machine”.

Although the device was clearly designed as a means to obtain minimally disturbed synchronous cells, this is not the application that was eventually used in most studies, including those that yielded information on replication–division coordination. In the very early experiments, nucleic acid synthesis during the cell cycle was analyzed by collecting newborn cells released from the instrument, growing them synchronously, and then pulse labeling them with radioactive precursors. That approach was laborious and resulted in cells that showed some evidence of growth disturbance. The growth disturbance turned out to be related to incorrect media preparation, which was soon corrected, but that issue forced me to rethink the whole idea of synchronous growth studies. Then, in 1966, I realized that cell cycle studies with the baby machine could best be conducted with minimal disturbance by performing the experiments in reverse. In this approach, the exponentially growing cells were pulse labeled just prior to attachment in the baby machine, and radioactivity of the newborn cells was determined as they were eluted. Each generation of newborn cells sequentially released during the growth and division of attached cells in the instrument were the daughters of the oldest cells, then the youngest cells in the original exponential phase culture, respectively. Thus, the radioactivity of newborn cells reflected the reversed incorporation during the cell cycle. From this point on, cell cycle studies were stunningly easy to perform and the data produced were very clear. After many years of trying, it only took a few weeks to determine the pattern of DNA replication during the cycle of slow-growing *E. coli* B/r A cells with that procedure using radioactive thymidine [13]. In essence, our work was based on a comment originally made by David Friefelder in 1963 plus the realization that we must conduct the experiments in the most effective, easiest manner.

The idea to use the baby machine in reverse stemmed directly from an experiment Steve Cooper and I conducted while we were both postdocs working with Ole Maaløe in Copenhagen in 1963–1964. We were interested in the process of chromosome segregation and decided that the baby machine technique would be an ideal method to determine if there were nonrandom aspects of chromosome segregation. So, we performed an experiment such as the type described above with radioactive thymidine and observed that the radioactivity per released newborn cell decreased essentially two-fold in each generation grown with the instrument, suggesting the random segregation of the labelled DNA strand between attached and released daughter cells. Cooper and I did not think our finding was very interesting at the time, and we both moved on; Cooper took up another postdoctoral position, and I took up a position in what is now the Roswell Park Comprehensive Cancer Center. Then, about two years later, upon belatedly arriving at the proper interpretation of an experiment of this type, I wrote to Cooper to tell him about it. He was euphoric, and it just happened that he was finishing a postdoctoral position and was moving to a new facility that was not quite ready for his arrival. As a result, we thought it would be a great idea for us to work together in my laboratory at Roswell Park for a while and investigate the cell cycle properties of rapidly growing cells. Our work progressed rapidly because it only involved loading the device with labelled cells every day and simply measuring the radioactivity of the cells that poured out. Resultant data showing radioactivity per newborn cell versus elution time required analysis [14,15,16], but even that task was simplified by the critical prior work of Schaechter et al. [17], which showed us how to conduct the experiments, and the findings by Sueoka and colleagues of the existence of multi-forked chromosome replication [18].

### 2.2. Mammalian Cells

Many years later, the baby machine concept was extended to mammalian cells. It was found to be applicable to cells that did not normally adhere to or spread on surfaces such as lymphoid cell lines [19]. The performance of the technique, in terms of both the purity of the released newborn cells and the longevity of their production, proved to be superior to the technique used for bacterial cells. In fact, it was possible to operate a rather complex mammalian cell baby machine for at least five generations with minimal degradation of the purity or concentration of newborn cells, thus achieving a nearly steady-state growth condition [20]. Unfortunately, mammalian cell cycle studies rarely employ techniques designed to produce minimally disturbed cells, such as the baby machine or mitotic shake-off [21]. Although the baby machine technique is very simple in principle, it can be labor-intensive. During operation, samples of newborn cells must be collected over lengthy periods of time before the actual experiment can begin.

Many mammalian cell cycle experiments employ an inhibitor to align cells at specific stages in the cycle [21]. These procedures have the advantage of being easier to perform, while producing considerably more aligned cells. A frequently used technique involves treatment with inhibitors of DNA replication, which are intended to align cells at the beginning of S phase, followed by the initiation of inhibition to produce synchronized growth. It has been suggested that this treatment produces synchronized cells that the reflect processes of the normal cell cycle [22]. That argument depends on the definition of S phase. If it is defined as merely the period of chromosomal DNA synthesis, such as the *C* period in *E. coli*, then the cells would certainly be aligned at the start of S and reflect the processes associated with that alignment. However, if the S phase is defined as a stage or interval of the cycle, then all aspects of that interval would not be aligned. Thus, if there were unique events in the cycle interval during which chromosome replication took place that were not governed, in some fashion, by chromosome replication itself, those events would not be aligned or detected. This is why Cooper and I decided to define the *E. coli* cell cycle in terms of the time periods *C* and *D*, rather than phases such as S and possibly G2 [15,16]. *C* and *D* can only be considered to define phase s in the cycle when the doubling time is equal to or longer than (*C + D*).

## 3. Cell Age at the Initiation of Chromosome Replication

### 3.1. E. coli K-12 and B/r

**Figure 1:**
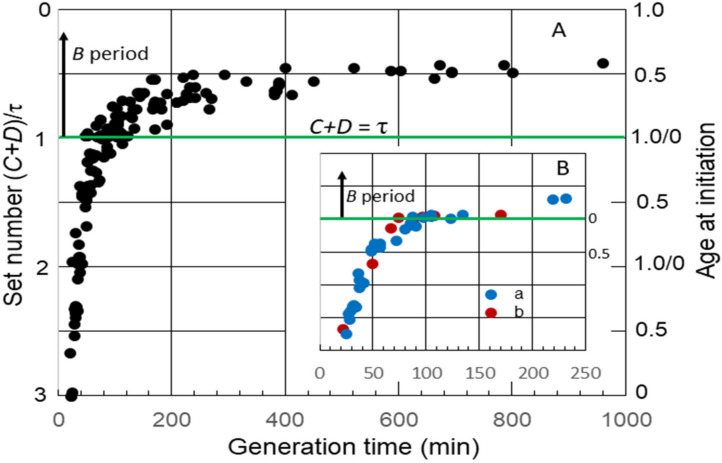
Set numbers and cell ages at the initiation of chromosome replication in *E. coli* growing with various generation times. (**A**) Collation of both set numbers and ages at the initiation of replication for seven strains of *E. coli* K-12 and three strains of B/r [23,24,25,26,27]. The green horizontal line in this and the subsequent figures shows age at initiation of replication when *C + D* is equal to the generation time, i.e., the initiation of replication at division in daughter cells containing a single chromosome. At longer generation times, there is a gap between division and initiation, relating to the *B* period above the green line. (**B**) Age at the initiation of replication versus generation time for: a: K-12 NJ24 [26] and b: K-12 GM1655 [27].

The average durations of *C* and *D*, as well *B*, the time between cell division and the initiation of replication by the single chromosome in slow-growing cells, have been measured in numerous strains of *E. coli*. I have used some of these data to examine the timing of the initiation of chromosome replication as a function of generation time (τ). Although this may be an unusual method for analyzing the durations of cell cycle periods, it yields interesting information that might not be obvious otherwise. This was accomplished by calculating the “set number”, defined as the generations between the start of a round of replication and the division after the end of that round [16], given by (*C + D*)/τ. The set numbers were then used to determine average age at initiation (a_i_) by subtracting the fractional part of the set number from 1.0 [16]. Figure 1A shows a collation of calculated set numbers and average ages at the initiation of replication for several K-12 and B/r strains grown in batch and chemostat cultures at 37 °C using data for *C* and *D* determined by flow cytometry [23,24,25,26,27]. As expected, the set numbers, and thus, the ages changed continuously throughout the period of rapid growth when the generation time was less than (*C + D*) for all strains, and a *B* period appeared when it was longer than (*C + D*). During very slow growth, there was minimal change in age at the initiation of replication. An interesting observation is that the initiation age immediately after τ = *C + D* varied considerably in different strains. (*C + D*) increased in all strains after τ became longer than the duration of (*C + D*) during rapid growth, but this occurred at different rates. In some strains, it increased in correlation with τ, such that the initiation age remained close to 0 for a time before a significant B period appeared (Figure 1B, inset). Ascribing any significance to the strain differences in durations of (*C + D*) requires additional data. Fortunately, some early studies with strain B/r are useful in this regard.

### 3.2. Age at the Initiation of Chromosome Replication in E. coli B/r A, F and K

As indicated above, the baby machine technique was developed using *E. coli B/r* A as the experimental organism. Although we were eventually able to use K-12 strains in the technique [28], the original procedure functioned poorly with K-12. If I had not stumbled upon B/r A in the beginning, the technique might not have been developed, and our work might have taken an entirely different direction. For the first 15 years, all of our work was performed exclusively with B/r A, including the work Cooper and I published in 1968 [14,15,16]. Eventually two additional B/r strains were used, B/r F from Ole Maaløe via James Friesen, and B/r K from Herbert Kubitschek. My aim in the remaining sections of this review is to summarize some of the interesting findings reported over the years using strain B/r and to introduce the possibility that continuing investigations with this strain might prove to be enlightening.

**Figure 2:**
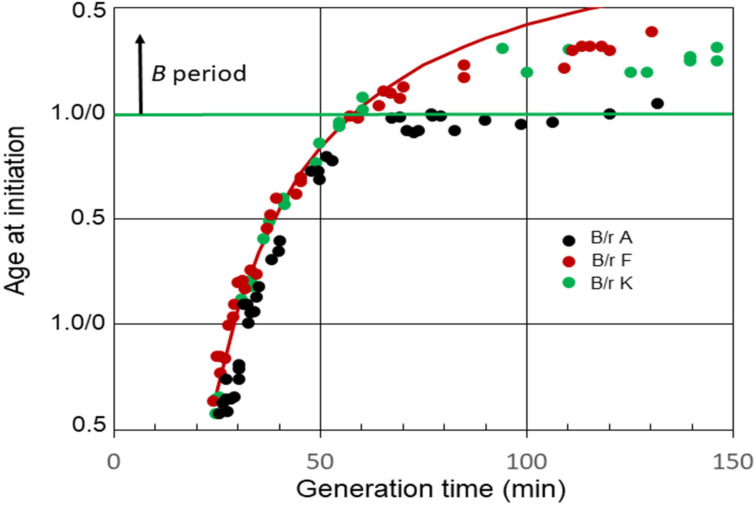
Cell age at the initiation of chromosome replication in *E. coli* B/r A, F, and K growing with various generation times. The red curve is theoretical age at the initiation of replication for B/r F, assuming (*C + D*) is constant and equal to 58 min.

The relationships between average ages at the initiation of chromosome replication and generation times for *E. coli* B/r A, F, and K observed in our experiments [29] are shown in Figure 2. *B* periods began to appear in strains B/r F and K when the generation time became equal to (*C + D*) min, or shortly thereafter. On the other hand, a *B* period was not detected in B/r A, at least between the generation times of 65 and 120 min. During this interval, it appeared that age at initiation remained basically constant just before cell division, at or near the end of the *D* period, meaning that (*C + D*) and τ were increasing at nearly the same rate.

The average *C* period at 37 °C was found to be 42 min during rapid growth (τ ≤ *C + D*) for all three strains [29]. On the other hand, the *D* periods differed, with the average *D* periods equaling 22, 16, and 14 min for B/r A, F, and K, respectively. The red curve in the figure shows the theoretical age at the initiation of replication for B/r F if (*C + D*) was constant at 58 min (*C* = 42 min; *D* = 16 min) at all the generation times. It is evident that the measured values for (*C + D*) in B/r F were constant until the generation time equaled (*C + D*). Thereafter, (*C + D*) began to increase, since the measured initiation ages lie below the curve, but it increased at a slower rate than the generation time did, unlike B/r A. In the same analysis, (*C + D*) also appeared to be constant in B/r A and K during rapid growth, but the generation time at which (*C + D*) began to increase was less certain. It is possible that (*C + D*) always begins to increase once the generation time is longer than the duration of (*C + D*) during rapid growth, but that is unclear from these data. The important point to note here is simply that B/r A differs from F and K during slow growth.

**Figure 3:**
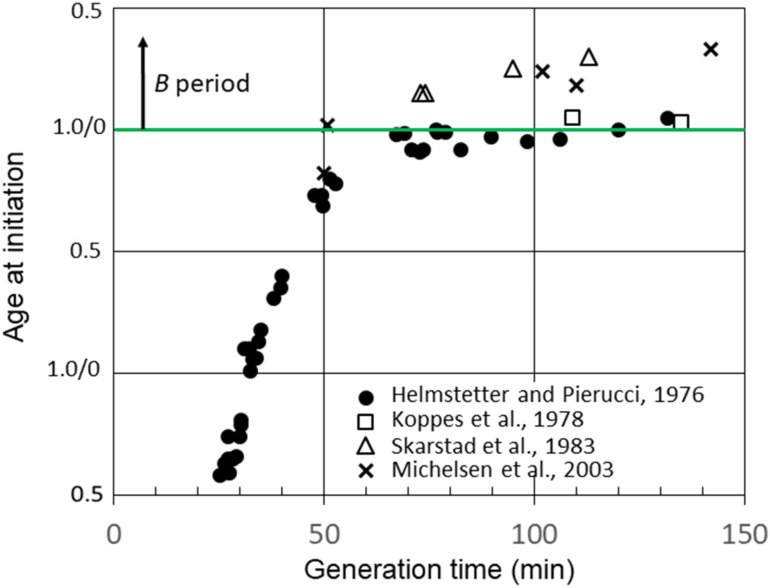
Cell age at the initiation of chromosome replication versus generation time for *E. coli* B/r A determined from measurements of *B*, *C,* and *D* durations in batch cultures by Helmstetter and Pierucci [28], Koppes et al. [30] and Michelson et al. [26], and in chemostat cultures by Skarstad et al. [23].

Figure 3 shows additional calculations of the average age at the initiation of replication in strain B/r A using data for *C* and *D* determined by researchers in other laboratories [23,26,30]. Calculations of the initiation age using data reported by Skarstad et al. [23] and Michelsen et al. [26] yielded values for slow-growing B/r A, which were very similar to those for F and K, as shown in Figure 2, and very different from our findings for B/r A; in other words, there was a significant early *B* period. However, another set of experiments performed with B/r A by Koppes et al. [30] were more consistent with our findings. As seen in Figure 3, they reported *B* periods in B/r A of 5 min and 3 min at generation times of 109 min and 135 min, respectively. They also found a *B* period of 39 min for B/r K growing with a generation time of 100 min, which is in basic agreement with our findings for this strain (Figure 2). The explanation for this odd disparity seen only with B/r A remains unknown, but it does not appear to be related to the techniques employed or the carbon sources used in the culture media since there were no consistent differences. The one consistent difference may relate to the culture media components other than carbon sources. The studies that found a small or no *B* period when the experiment was performed in C medium, which consists of M9 salts plus MgSO_4_ [29]. The experiments detecting significant *B* periods in B/r A when the experiment was performed in either M9 or AB medium, which contain additional salts, such as CaCl_2_ and FeCl_3_ [31]. Possible implications of these findings are described in Section 4.

### 3.3. Effects of Protein Synthesis Inhibition on Initiation of Replication

**Figure 4:**
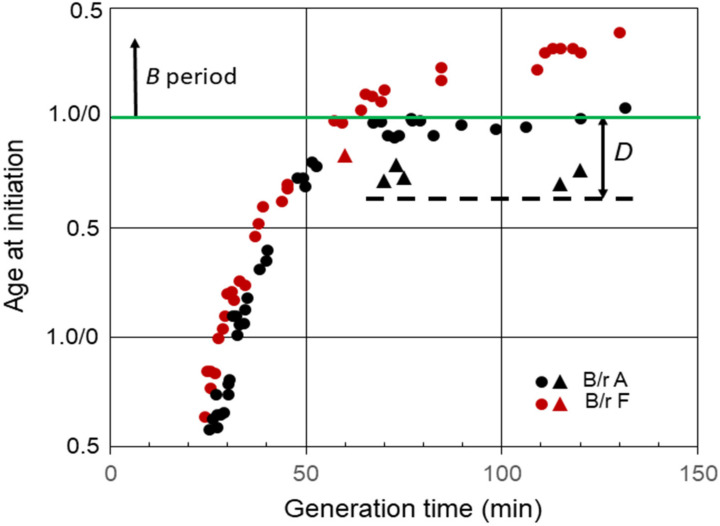
Effect inhibition of RNA and protein synthesis on the initiation of replication in slow-growing B/r A and F. The triangles indicate the ages of the youngest cells that were capable of progressing to initiation of replication in the presence of chloramphenicol or rifampicin plus chloramphenicol. The dashed line shows the approximate cell age at the start of the *D* period during slow growth of B/r A.

Another interesting property of *E. coli* B/r grown in C medium relates to the response of slow-growing cells to exposure to chloramphenicol or chloramphenicol plus rifampicin [32,33]. When the inhibitors were added to B/r A cells growing with generation times between approximately 60 and 120 min, that is, cells that normally initiated replication with two chromosomal origins during the *D* period, the cells continued to progress to initiation of replication. For example, when chloramphenicol was added to B/r A cells growing with a doubling time of 120 min, the cells continued to progress to initiation of replication for over 30 min (Figure 4). The triangles in the figure indicate the ages of the youngest cells that were able to continue to the initiation of replication in the presence of inhibitors. The cells that initiated replication were the only cells that divided between the two origins. However, division per se was not required since initiation of replication also takes place in the presence of penicillin [33]. This phenomenon was only observed in slow-growing cells that normally initiated during the *D* period with two origins. However, it was not unique to B/r A because when chloramphenicol and rifampicin were added to B/r F cells growing with a generation time of 60 min, i.e., cells that normally initiated toward the end of *D* [32], they also continued to initiate, but for only 10 min (Figure 4), presumably because they only divided for 10 min. If this behavior is true for all strains of *E. coli*, then determinations of *C*, as well as the average number of replication origins per cell and (*C + D*), in strains growing with generation times between *C* min and (*C + D*) min obtained by measuring the extent of DNA replication in the presence of rifampicin or chloramphenicol could yield erroneously large values. This would be especially true for cells growing with generation times slightly shorter than (*C + D*).

### 3.4. Relationship between Durations of C and D

**Figure 5:**
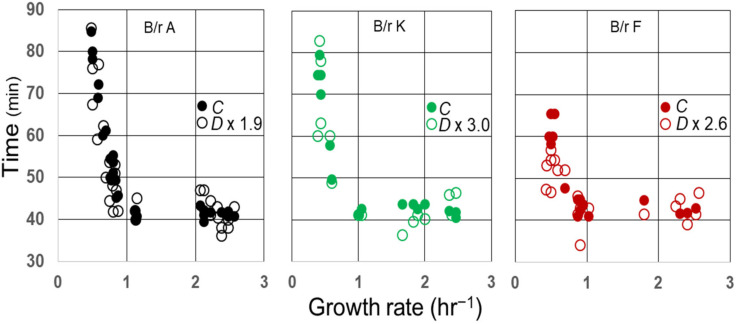
Correlation between *C* and *D* in *E. coli* B/r as a function of growth rate. Measurements of *C* and *D* and the average *C/D* ratios during rapid growth [τ ≤ (*C + D*)] were taken from Helmstetter and Pierucci [29] and used to plot *C* (closed circles) and *D* × *C/D* (open circles) versus growth rate.

It is well known that the average *C* period has been found to be constant in *E. coli* growing with generation times less than 60–70 min at 37 °C [26]. In *E. coli* B/r, the average duration of (*C + D*) has also been found to be indistinguishable from the constant during rapid growth [29], and that may also be true for some K-12 strains [27]. As a consequence, the average ratios of *C/D* during rapid growth of B/r A, F, and K are 1.9, 2.6, and 3.0, respectively [29]. To examine whether this relationship between *C* and *D* might continue during slow growth, the measured values for *D* during the slow growth of three strains (29) were multiplied by their respective ratios during rapid growth and compared to *C*, as shown in Figure 5. Since the values for *D* × (*C/D*) appear to superimpose on the values for *C* in B/r A and K during slow growth, the ratios *C/D* seem to be invariant at all growth rates examined in these strains and probably in B/r F as well.

### 3.5. Relative Sizes of B/r A, K, and F

A final interesting aspect of the B/r strains relates to their sizes and shapes. Woldringh et al. [34] have shown that the shapes of newborn B/r A and K cells grown in C medium are very similar during rapid growth, but they diverge considerably in shape during slow growth. At slower growth rates, B/r A becomes more spherical, whereas B/r K maintains a rod-like shape. However, newborn cell volumes calculated using cell dimensions were found to be similar for all three B/r strains when they were grown at the same rate. Referring back Figure 2, since in C medium B/r A initiates replication at an earlier age than B/r F or K do at all growth rates, if newborn cells have similar volumes, the cell volume per chromosomal origin at the initiation of replication must be smaller in A than those in F or K. Indeed, that is what has been reported [30], and it can be found using known values of (*C +D*) to calculate cell volume at the time of the initiation of replication from the cell dimension measurements. Perhaps the difference in initiation volumes is related to the different shapes of strains during slow growth. However, that cannot explain the differing initiation volumes during rapid growth. When B/r A and K were grown rapidly in C medium at a rate of about 2.5 doublings/hr, the newborn cells were very similar in shape [34]. Thus, at this rapid growth rate, the newborn cell volumes, cell shapes, *C* period durations (42 min), and times for cell constriction (T ≈ 10 min [34]) were found to be essentially the same. The one consistent difference is in the *D* duration, along with a concomitant difference in initiation age. These findings raise questions regarding the relationship between initiation timing and *D* period duration.

## 4. Discussion

The preceding information suggests, but it certainly does not prove, that the comparative cell cycle properties of *E. coli* B/r A, F, and K may depend on the culture conditions. In one set of conditions, the findings, albeit limited, suggest that the three strains may be very similar at all growth rates with regard to the newborn cell size, the cell age/volume at the initiation of chromosome replication, the durations of *C* and *D* periods, and the cell shape. Under an alternative culture condition, during growth in C medium, B/r F and K appear to maintain the same cell cycle properties, but the key properties of B/r A differ, namely, the initiation volume is smaller, and the *D* period is longer at all growth rates. Given this striking difference in these properties in one strain grown at the same rate in different media, it is interesting to enquire about the connection between cell size at the initiation of chromosome replication and *D* duration. There are a few possible explanations. The first and least interesting one is the possibility that the observed differences arise due to differing techniques and procedures used in different laboratories. Although this explanation seems to be unlikely, it would be decidedly more reassuring if these studies were performed simultaneously by one research group.

A likely scenario is that the longer duration of *D* in B/r A grown in C medium is a direct consequence of the smaller size at initiation, resulting in the initiation of replication at an earlier cell age. There are several observations that, taken together, support this conclusion. Based on current views of the cell cycle and assuming that the cell volume and cell mass are interchangeable, if the cell volumes (V) and *C* periods are the same in B/ r A and K grown at the same rate, but the initiation volume (V_i_) of B/ r A is smaller, then B/r A must, out of necessity, have a longer *D* period since V_i_ = V/ln2e^(*C+D*)/τ^ [35]. Additionally, the duration of *D* seems to be uninfluenced by the time for cell constriction and the T period, since during rapid growth, T averages about 10 min in both B/r A and K [34], whereas the average *D* periods are 22 and 14 min, respectively [29]. The T period also increases considerably more in B/r A than it does in K during slow growth [34], but in spite of that, the *D/C* ratio remains essentially unchanged in both strains. As plausible as it may be that the initiation size could set the *D* duration, it does not account for the smaller initiation volume in this B/r strain in one culture medium. The explanation could simply be that there is an increased relative concentration of DnaA or DnaA-ATP in B/r A when it is grown in C medium, but information on that issue is not currently available.

Another scenario is that the smaller initiation size is a direct consequence of the longer *D* in B/r A grown in C medium. On the surface, the involvement of *D* in the process of replication initiation would be surprising since it has been reported that increasing the duration of *D* by altering the cell shape does not alter the initiation mass [36,37,38]. However, in the studies on *E. coli* B/r referenced here, the cell shapes during rapid growth were basically the same, in spite of the differing *D* durations. On the other hand, the observation of a dramatic shape change in B/r A as it grew slower in C medium might suggest that some aspect of surface growth could be aberrant in B/r A at all growth rates, possibly yielding a longer *D* period when it is grown in C medium. How might a longer *D* result in a smaller initiation volume? We know that B/r cells that were normally initiated in *D* were able to progress to the initiation of replication in the absence of protein synthesis as long as they progressed toward division between two chromosomal origins. That phenomenon cannot be a direct consequence of the increased relative concentration of DnaA protein that has been observed during slow growth [27,39,40] since initiation of replication during the inhibition was not seen in cells that normally initiated replication just before or just after the *D* period. It seems to be associated with some specific aspect of *D*, such as envelope formation/division progression. There is evidence that domains of acidic phospholipids may form during *D* [41], which might be capable of DnaA-ADP-to-DnaA-ATP conversion and the consequent initiation of replication. Perhaps this process could augment the progression to initiation during the cycle of cells with longer *D* periods, resulting in a smaller initiation size.

Lastly, since cell age at the initiation of replication is relatively unchanged, at or near cell division, in B/r A for an extended period once the generation time becomes longer than the average value of (*C + D*) during rapid growth (Figure 2), as may be the case with other strains as well (Figure 1), a question arises as to the size at initiation during that interval. In the case of B/r A, cell dimensions determined by Woldringh et al. [34] indicated that the initiation volume decreased as the generation time increased from 65 to 120 min. This is also a time when *D* becomes increasingly longer which, as discussed above, could be a potential explanation for the smaller initiation size in these cells. Alternatively, the observed increase in relative DnaA concentration during slow growth [27,40] could also be a reasonable explanation for the decreasing initiation size. Even if these conjectures have some level of validity, an attempt to explain the basis for the apparent lack of significant change in cell age at the initiation of replication during an interval of slow growth would be premature in the absence of additional information.

Much of the preceding data are speculative and may turn out to be of minimal significance. What is significant is that I feel it is possibility that a more comprehensive analysis of the properties of *E. coli* B/r, and especially the seemingly unusual B/r A, might yield some interesting new information on cell cycle controls, and hence, the validity of current cell cycle control models.

## Data Availability

A video with additional information can be viewed at https://drive.google.com/file/d/1r7mrP6E46-fam7wKsjHYritBGWMgJhJC/view.
